# Insulin-like growth factor-1 overexpression increases long-term survival of posttrauma-born hippocampal neurons while inhibiting ectopic migration following traumatic brain injury

**DOI:** 10.1186/s40478-020-00925-6

**Published:** 2020-04-10

**Authors:** Erica L. Littlejohn, Danielle Scott, Kathryn E. Saatman

**Affiliations:** 1 Spinal Cord and Brain Injury Research Center, Department of Physiology, University of Kentucky, B473 Biomedical & Biological Sciences Research Building (BBSRB), 741 South Limestone St, Lexington, KY 40536-0509 USA; 2 Department of Cellular and Integrative Physiology, University of Texas Health Science Center at San Antonio, 7703 Floyd Curl Drive, San Antonio, TX 78229-3901 USA; 3 Department of Physiology, College of Medicine, University of Kentucky, Lexington, KY 40536 USA

**Keywords:** Cognitive flexibility, Controlled cortical impact, Dentate gyrus, Gliogenesis, Granule cell layer, Neurogenesis, Reversal learning

## Abstract

Cellular damage associated with traumatic brain injury (TBI) manifests in motor and cognitive dysfunction following injury. Experimental models of TBI reveal cell death in the granule cell layer (GCL) of the hippocampal dentate gyrus acutely after injury. Adult-born neurons residing in the neurogenic niche of the GCL, the subgranular zone, are particularly vulnerable. Injury-induced proliferation of neural progenitors in the subgranular zone supports recovery of the immature neuron population, but their development and localization may be altered, potentially affecting long-term survival. Here we show that increasing hippocampal levels of insulin-like growth factor-1 (IGF1) is sufficient to promote end-stage maturity of posttrauma-born neurons and improve cognition following TBI. Mice with conditional overexpression of astrocyte-specific IGF1 and wild-type mice received controlled cortical impact or sham injury and bromo-2′-deoxyuridine injections for 7d after injury to label proliferating cells. IGF1 overexpression increased the number of GCL neurons born acutely after trauma that survived 6 weeks to maturity (NeuN+BrdU+), and enhanced their outward migration into the GCL while significantly reducing the proportion localized ectopically to the hilus and molecular layer. IGF1 selectively affected neurons, without increasing the persistence of posttrauma-proliferated glia in the dentate gyrus. IGF1 overexpressing animals performed better during radial arm water maze reversal testing, a neurogenesis-dependent cognitive test. These findings demonstrate the ability of IGF1 to promote the long-term survival and appropriate localization of granule neurons born acutely after a TBI, and suggest these new neurons contribute to improved cognitive function.

## Introduction

TBI is the leading cause of death among people under 40 [1, 2]. Survivors of TBI often suffer from persisting learning and memory deficits [3–6], which may be related to the brain’s limited capacity to repair and replace lost neurons.

Throughout adulthood, neural stem cells in the subgranular zone (SGZ) of the hippocampal dentate gyrus (DG) proliferate and differentiate into new principal granule neurons of the granule cell layer (GCL) [7]. During normal adult neurogenesis, as many as half of all newly born neurons undergo apoptosis within 4 weeks of proliferating [8–11]. Surviving neurons form synapses to functionally integrate into the local hippocampal circuitry [12, 13]. Adult-born neurons have been shown to contribute to contextual fear memory and extinction [14, 15], spatial memory [16, 17], pattern separation [18], and cognitive flexibility [18–21].

Trauma has a pronounced impact on immature hippocampal neuron survival and development. Contusive brain injury results in the selective death of immature neurons in the GCL within days [22–24]. Proliferation of neural stem cells in the SGZ is concomitantly stimulated, resulting in recovery of immature neuron numbers within 1–2 weeks [23–26]. While a considerable subset survive to maturity [27–32], posttrauma-born hippocampal neurons develop with atypical dendritic arbors [25, 26, 33, 34], which may alter synapse-dependent maturation and survival [35–37]. Furthermore, TBI appears to stimulate outward migration of new neurons within the GCL [33, 38, 39] and may foster ectopic migration of a small percentage of newly born neurons from the GCL into either the molecular layer (ML) or hilar layer (HL) [34, 39, 40]. Mislocalization and aberrant development may interfere with the function and survival of neurons born following TBI.

Insulin-like growth factor-1 (IGF1) is a potent growth factor that is expressed at high levels during neuronal development and at lower levels in the adult CNS [41, 42]. IGF1 supports neurite outgrowth [43], is required for appropriate migration of neural progenitors during development [44], and promotes newborn neuron survival [45]. In the context of injury, we have shown that IGF1 increases the number of immature neurons born after trauma and restores their dendritic arborization to the levels of uninjured controls [26, 46]. Additionally, IGF1 has been shown to attenuate acute posttraumatic cognitive dysfunction [47–49].

Here, we utilized a transgenic mouse model in which conditional overexpression of IGF1 by astrocytes results in increased hippocampal levels of IGF1 acutely after contusive brain injury [48]. At 6 weeks post-injury, a time point beyond the window of maturation-dependent survival of adult-born DG granule cells [9], we examined the effects of IGF1 overexpression on the survival and localization of matured granule neurons born within the first week after TBI. IGF1 overexpression increased the number of posttrauma-born neurons that survived to end-stage maturity and reduced ectopic migration. IGF1 overexpression improved radial-arm water maze (RAWM) reversal learning after the 6-week period corresponding to enhanced stable neurogenesis, suggesting that IGF1 supports long-term cognitive recovery after TBI.

## Materials and methods

### Animal care

Transgenic mice with astrocyte-specific conditional overexpression of IGF1 were described previously [48, 50]. The mice were bred in-house by crossing heterozygous tTA^GFAP^ mice with heterozygous IGF1^pTRE^ mice, yielding double transgenic mice carrying both transgenes (tTA^GFAP^/IGF1^pTRE^) [26, 48]. IGF1 double transgenic mice (IGFtg) and their wildtype (WT) littermates were fed doxycycline supplemented chow (200 mg/kg) to suppress IGF1 expression from birth until 2 weeks prior to surgery, after which they received standard chow. The mice were provided with food and water ad libitum at the University of Kentucky Medical Center animal vivarium. They were housed in temperature-controlled rooms (23 ± 2 °C) with a 14/10-h light/dark cycle.

### Controlled cortical impact injury

Isoflurane-anesthetized mice aged 13.3 ± 0.5 weeks old (*n* = 55) received a 5-mm diameter craniotomy over the left parietal cortex. For sham injury, a cranioplasty was then affixed to the skull to protect the exposed dura and the incision was closed. For controlled cortical impact (CCI) injury, an impactor with a rounded 3 mm diameter tip was used to deliver a rapid (3.5 m/s) impact to the intact dura, as previously described [26, 48]. For immunohistochemical analysis of hippocampal neurogenesis, a cohort of WT (5 female, 7 male) and IGFtg (6 female, and 5 male) mice were randomized for sham injury (3 per genotype) or CCI (8–9 per genotype) delivered using a pneumatically driven impactor device (Precision System Instruments). A second cohort designated for behavioral analysis consisted of WT (6 female, 7 male) and IGFtg (3 female, 8 male) mice that received CCI using a stereotaxic electromagnetic impactor (Leica Biosystems). As a behavioral analysis reference group, a third cohort of WT mice (3 female, 5 male) received sham injury. Impact depths were set for each device (0.8 mm for pneumatic, 1.1 mm for electronic) to produce comparable injury severity across the two cohorts as determined, prior to the onset of these studies, by the size of the cortical contusion.

### BrdU administration

Beginning 24 h after CCI or sham injury each animal received two intraperitoneal injections of 5-Bromo-2′-deoxyuridine (BrdU; 50 mg/kg in saline; Fisher Scientific) at an 8 h interval each day until 7 days post-injury (dpi). Proliferating cells were labelled from one to 7 days after injury to capture the majority of the proliferative phase triggered by TBI [23, 26].

### Histology and immunohistochemistry

Animals were deeply anesthetized by sodium pentobarbital (Fatal-Plus Solution, Vortech Pharmaceuticals) and transcardially perfused with heparinized saline followed by 10% buffered formalin at 42 dpi. Brains were removed 24 h after post-fixation in 10% formalin, cryoprotected using 30% sucrose solution, and snap frozen in cold isopentanes (≤ − 25 °C). Frozen brains were cut in a coronal plane at 40 μm thickness.

Immunofluorescence was performed using published protocols for free-floating sections [26, 48] on three tissue sections selected at 400 μm intervals spanning the injury epicenter (between − 1.5 to − 2.5 mm bregma). To expose BrdU epitopes, the tissue was incubated in 2 N HCl (Fisher Scientific) at room temperature with agitation for 1 h, followed by 100 mM borate for 10 min to neutralize residual HCl. The tissue was rinsed overnight in TBS at 4 °C prior to a 30 min room temperature incubation in TBS with 0.1% Triton-X-100 and 5% Normal Horse Serum (NHS). Primary antibodies were diluted in TBS with 0.1% Triton-X-100 and 5% NHS and tissue was incubated overnight at 4 °C with agitation. Primary antibodies used were anti-NeuN (rabbit, 1:500, Millipore Sigma), anti-GFAP (rabbit polyclonal, 1:1000, Millipore Sigma), anti-Iba1 (rabbit polyclonal, 1:1000, Wako), and anti-BrdU (rat monoclonal, 1:1000, Abcam). Secondary antibodies were conjugated with Alexa-488, Cy-3 or Alexa-594 (Invitrogen). The omission of primary antibody served as a negative control. Sections damaged during processing were omitted from analysis for one mouse.

### Image acquisition

Images were taken on a confocal epifluorescence microscope (C2 Tie Nikon confocal microscope) capturing the DG for each section per animal. BrdU+ and NeuN+BrdU+ cells in the densely packed GCL of the hippocampus ipsilateral to impact were imaged at 100x magnification under oil and at 0.75 μm step intervals through a 25 μm depth. Maximum intensity projection images of glia labelled with BrdU in the ML were generated from confocal images of the DG taken at 20x magnification at 1.5 μm step intervals through 20 μm of each section.

### Cell quantification

The region of interest (ROI) area (mm^2^) and section thickness (mm) were auto-detected by NIS-Elements image analysis software using DAPI counterstain and confirmed by an observer and used to determine ROI volume (mm^3^). Cell counts were manually quantified in 3D using NIS-Elements image analysis software by an observer blinded to genotype and injury group. Absolute cell counts were normalized to the ROI volume (mm^3^) and expressed as density (count/mm^3^). To evaluate the effect of IGF1 on adult-born neuronal survival in the GCL, the numbers of BrdU+ and NeuN+BrdU+ cells in the GCL, ML, and HL were counted. Cells were determined to reside in the GCL if they were within one cell distance (0-10 μm) of the GCL border and the soma was adjacent to other NeuN+ cells along the border [34]. To determine the position within the GCL of neurons that were generated within the first week of injury (NeuN+BrdU+), the location of the center of the cell in relation to the GCL/HL border was determined as previously described [33, 51] and annotated using NIS-Elements, and cells were binned into the inner 1/3 (iGCL) or outer 2/3 (oGCL) of the GCL. As principal granule cells are the only type of neuron generated by adult neurogenesis in the DG, NeuN+BrdU+ neurons located in either the HL and ML were considered ectopic [7, 52]. To quantify the fraction of proliferated neurons localized ectopically, the combined number of proliferated neurons localized to the ML and HL were normalized to the total number of proliferated neurons in the DG (i.e. (ML + HL)/(ML + HL + GCL)).

Injury induces glial activation and proliferation within the DG [23, 32, 53–55]. To determine if IGF1 increased proliferation of glial cells, BrdU+, Iba1+BrdU+, and GFAP+BrdU+ cells were counted in the ML and normalized to volume. To determine if IGF1 overexpression altered the pattern of glial representation after injury, areas of GFAP and Iba1 immunoreactivity within the ML were quantified and normalized to the ML ROI area.

### Radial arm water maze (RAWM)

The water maze behavior assessment describes a spatial learning test in which animals are trained to locate a fixed platform hidden beneath the surface of a pool. The water component serves as an aversive motivator that encourages rodents to escape quickly. Decreased exploration of areas that do not contain the platform over contiguous trials indicates learning. The reversal paradigm introduces a novel platform location after animals have learned to find the original location. How quickly rodents decrease preference for the old location and replace it with a novel location is an indicator of cognitive flexibility [20, 56]. Cognitive flexibility has been linked to an increase in the number of mature adult-born neurons [20].

To measure spatial reference memory and learning, mice were tested using an established 2-day RAWM acquisition protocol [57, 58] beginning 39 dpi by an observer blinded to animal genotype. The six-arm maze had an arm length of 30 cm and a common circular swim diameter of 40 cm within a 100 cm diameter pool. The pool was filled with 20–21 °C water, made opaque using tempura paint (Rich Art Co.), to a level approximately 2 cm above a clear (hidden) circular platform. The platform was placed at the end of an arm approximately 7 cm away from the side and back walls. During visible trials, a cue was placed on the edge of the platform. The pool was enclosed by a black curtain on which four unique geometric extra-maze visual cues were affixed.

On day 1 (acquisition day), mice were trained over 12 trials to identify the platform location, alternating between visible and hidden platform trials [58]. Trials 13–15 on day 1 consisted of 3 hidden platform trials, yielding a total of 15 trials. On day 1, mice unable to locate the platform within 60 s were gently guided to it. On day 2, reference memory was tested using 15 hidden platform trials. Mice received reversal training on the third day of testing, during which the platform was moved to a novel location, at least 2 arms away from the original location [57, 59]. On day 3, mice received 15 hidden platform trials. Training was conducted in 5 blocks of 3 trials each day of testing (days 1–3). Mice were tested in groups of 4–5 and never entered the same start arm as the previous trial. A mouse was considered to have entered the arm if its whole body crossed the threshold of the arm. Animals received an error point if they entered the wrong arm or did not make an arm choice for more than 15 s. Entries into the goal arm were not counted as errors even if the mouse did not locate the platform. Mice that have learned the RAWM exhibit performance errors of 1 or less, averaged over three trials near the end of the second day [57]. Control (WT sham mice, *n* = 8) learned the RAWM test by the third block of day 2, averaging 1.4 ± 0.3 errors. Because over-training animals is taxing and could influence performance [57, 60], the first 3 blocks of hidden platform trials during acquisition days 1 and 2 were used for analyses. The error data is presented as the sum of the errors during hidden platform trials per block.

Upon completion of RAWM testing, visual and swimming deficits were evaluated using an open pool visible platform test [57]. The latency to find the flagged platform in an open pool divided into 4 equal quadrants was assessed over 5 blocks of three 60-s trials. The start position remained constant throughout the testing, while the goal quadrant varied for each trial. Animals were excluded from RAWM analysis for failing the visible platform test [57] by receiving a latency of > 20 s in the last block of the test and ≥ 45 s latency across all 15 open pool trials (1 WT and 1 IGFtg). Two visually competent mice were excluded from analyses for non-participation, failing to reach the platform in every trial during the 3 days of testing (1 WT and 1 IGFtg).

All trials were recorded with a digital camera using EthoVision XT 8.0 video tracking software (Noldus Information Technology). The error and latency to platform data were manually analysed offline by blinded observers. Activity heat maps were generated by video analysis using Anymaze software (Stoelting Company).

### Experimental design and statistical analyses

Data were acquired and assembled by an individual blinded to genotype and injury conditions. Statistical analyses and graph generation were performed using GraphPad Prism software 6.0. Cell counting and localization assays revealed no differences within groups as a function of the anterior-posterior coordinate of the sections; therefore, cell count data were summed across tissue sections. Cell counts and cell positioning data were analysed using a one-way analysis of variance (ANOVA), followed by post-hoc Bonferroni’s multiple comparison t-tests where appropriate. Post-hoc comparisons were limited a priori to four, to test for genotype effects across sham groups and across injured groups, and injury effects (sham versus CCI) for WT mice and for IGFtg mice. Where appropriate, Student’s T-tests were used to compare groups of two. RAWM (days 1–2) and RAWM reversal (day 3) data were analysed using a one-way ANOVA with repeated measures. Because the sham mice tested in the RAWM constituted a separate study and were not randomized with the WT and IGFtg CCI groups, the sham behavioral responses were not included in statistical analyses. Data are presented as mean + standard error of mean (SEM). For all comparisons *p* < 0.05 was considered statistically significant. Statistical analyses for data in all figures are presented in Tables 1 and 2.

**Table 1:** Summary of statistics for cell type and location of posttrauma-proliferated cells

Measure	Figure	One-way ANOVA	ANOVA p value	Bonferroni’s selected comparisons (*p* value)			
				WT vs IGFtg (Sham)	WT vs IGFtg (CCI)	Sham vs CCI (WT)	Sham vs CCI (IGFtg)
*BrdU density*							
GCL	1H	F(3, 19) = 6.394	0.0035	ns	ns	0.0349	0.0246
ML	2E	F(3, 19) = 7.972	0.0012	ns	ns	0.0049	0.0395
*GCL NeuN + BrdU+*							
Density	1I	F(3, 19) = 7.761	0.0014	ns	0.0034	ns	0.0228
Proportion	1J	F(3, 19) = 13.33	0.0001	ns	0.0001	0.0028	ns
*ML GFAP + BrdU+*							
Density	2F	F(3, 14) = 6.899	0.0044	ns	ns	0.0193	0.0330
GFAP area	2H	F(3, 13) = 7.579	0.0035	ns	ns	ns	0.0052
*ML Iba1 + BrdU+*							
Density	2G	F(3, 18) = 4.432	0.0168	ns	ns	0.0355	ns
Iba1 area	2I	F(3, 17) = 5.135	0.0104	ns	ns	ns	0.0143
*NeuN + BrdU+ localization density*							
iGCL	3C	F(3, 19) = 6.86	0.0025	ns	0.0080	ns	0.0267
oGCL	3D	F(3, 19) = 5.445	0.0071	ns	0.0158	ns	0.0513
*NeuN + BrdU+ localization proportions*							
oGCL	3E	F(3, 19) = 5.941	0.0049	ns	ns	ns	0.0476
ML + HL	3G	F(3, 19) = 9.179	0.0006	ns	0.0352	0.0024	ns

**Table 2:** Summary of statistics for radial arm water maze (RAWM) learning

Measure	Figure	Repeated measures 2-way ANOVA		***p*** value
RAWM Error Day 1–2	4A	Genotype	F(1, 18) = 0.612	0.4442
		Block	F(5, 90) = 3.023	0.0144
		Interaction	F(5, 90) = 0.994	0.6610
RAWM-R Error Day 3	4C	Genotype	F(1, 18) = 0.784	0.3874
		Block	F(4, 72) = 2.697	0.0373
		Interaction	F(4, 72) = 0.982	0.4228
Cognitive Flexibility: Time in old arm	4D	Genotype	F(1, 18) = 5.099	0.0366
		Block	F(4, 72) = 1.844	0.1298
		Interaction	F(4, 72) = 1.684	0.1631
Visible Platform Latency	4E	Genotype	F(1, 18) = 1.118	0.3044
		Block	F(4, 72) = 9.989	0.0001
		Interaction	F(4, 72) = 0.386	0.8180

## Results

### IGF1 overexpression enhances posttraumatic neurogenesis without increasing cellular proliferation

**Fig. 1:**
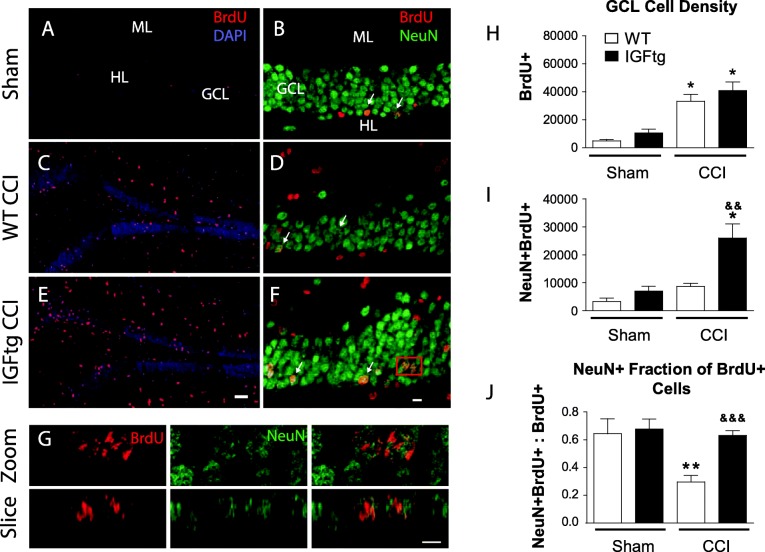
IGF1 overexpression enhances long-term posttraumatic neurogenesis and rescues trauma-induced reduction of differentiated mature neuron pool. Controlled cortical impact (CCI) enhanced proliferation in both wildtype (WT) and IGF1 overexpressing (IGFtg) mice. Cells dividing within 7 days following surgery were detected at 6 weeks after injury in the granule cell layer (GCL), molecular layer (ML) and hilus (HL) using bromodeoxyuridine (BrdU, red) immunolabelling of (**a**, **b**) sham controls, (**c**, **d**) injured WT, and (**e**, **f**) injured IGFtg mice. DAPI label is shown in blue. A subset of BrdU+ cells in the GCL colabelled with the mature neuron-specific protein, Neuronal Nuclei (NeuN, green) (**b**, **d**, **f**). Examples of colabelled cells are noted by arrows. (**g**) The phenotype of posttrauma-proliferated cells was confirmed by 3D reconstruction of confocal images, as demonstrated in high-magnification images for the cells in the red box in (**f**). The scale bar represents 50 μm in A, C and E, 10 μm in B, D and F, and 5 μm in G. (**h**) The density of BrdU+ cells (cells/mm^3^) within the GCL was increased in injured WT and injured IGFtg mice relative to their respective sham controls. (**i**) Following injury, IGF1 overexpression increased the density (cells/mm^3^) of surviving posttrauma-born neurons (NeuN+BrdU+) in the GCL. (**j**) Trauma reduced the proportion of proliferated progenitors that matured into end-stage neurons in WT mice but not in IGF1tg mice. Data are presented as mean + SEM; *n* = 3 sham /genotype, *n* = 8–9 CCI /genotype. One-way ANOVA, followed by Bonferroni’s selected comparisons post-hoc t-tests: *p < 0.05 and **p < 0.01 compared to respective sham group, and ^&&^*p* < 0.01 and ^&&&^*p* < 0.001 compared to injured WT

To determine the effect of IGF1 on long-term survival of cells that proliferate after TBI, we used BrdU to label cells dividing within the first 7 dpi and quantified the number of BrdU+ cells persisting until 42 dpi in IGFtg and WT mice. Proliferated cells were distributed throughout the DG and were more abundant in mice with TBI (Fig. 1a, c, e). Quantification in the GCL confirmed a significant increase in the density of acutely proliferated (BrdU+) cells that survived 6 weeks in both WT and IGFtg injured mice compared to sham controls (Fig. 1h). The injury-induced increase in proliferation within the GCL was equivalent in WT and IGFtg mice.

We previously showed that IGF1 stimulates an increase in numbers of immature neurons in the GCL early after CCI brain injury by promoting neuronal differentiation [26]. To determine if overexpression of IGF1 supported long-term survival of neurons born acutely after trauma, we co-labelled BrdU+ cells with NeuN (Fig. 1b, d, f, g) and quantified the density of proliferated neurons in the GCL at 42 dpi. In WT mice, brain injury did not result in a statistically significant increase in the density of NeuN+BrdU+ neurons in the GCL. In contrast, brain-injured IGFtg mice displayed a marked increase in NeuN+BrdU+ cell density when compared to either IGFtg shams or brain-injured WT mice (Fig. 1i). The density of surviving posttrauma-born neurons in brain-injured mice overexpressing IGF1 was nearly 3-fold higher than in injured WT mice, demonstrating that IGF1 stimulates stable hippocampal neurogenesis in the traumatically injured brain. In brain-injured WT mice, less than 30% of BrdU+ cells in the GCL expressed NeuN (Fig. 1j). IGF1 overexpression significantly increased the proportion of BrdU+ cells that exhibited a mature neuronal phenotype following injury to over 60%, restoring the balance of proliferated neurons a level equivalent to sham controls.

### IGF1 overexpression does not potentiate posttraumatic gliogenesis in the DG

**Fig. 2:**
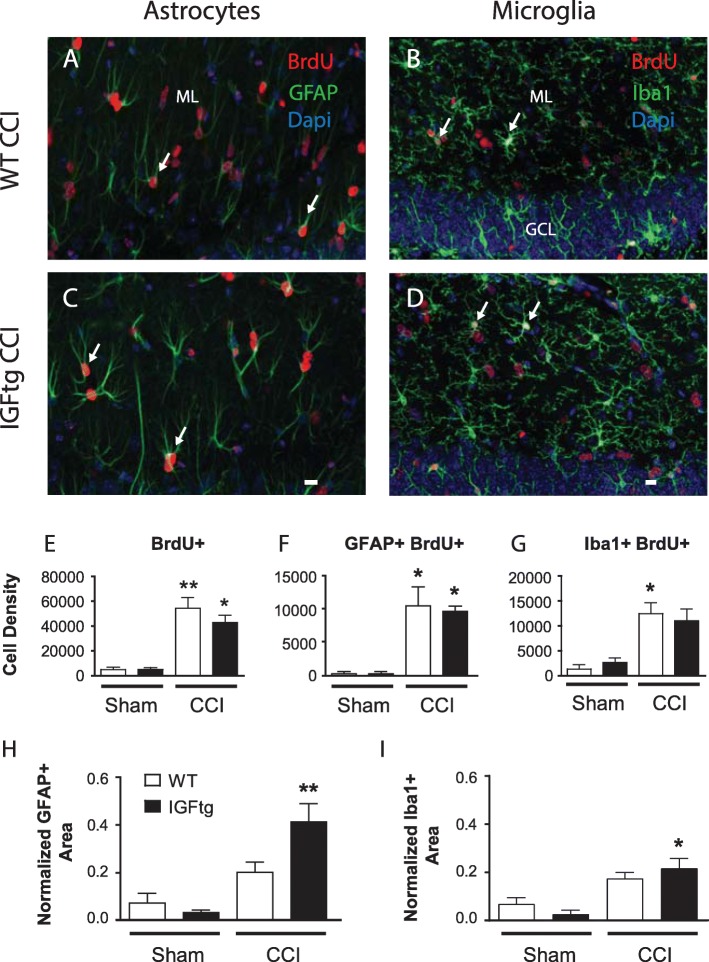
IGF1 overexpression does not potentiate posttraumatic gliogenesis in the dentate gyrus. Cells that proliferated during the first week after controlled cortical impact (CCI) detected using bromodeoxyuridine (BrdU, red) were colabelled with (**a**, **c**) the astrocyte marker, glial fibrillary acidic protein (GFAP, green) or (**b**, **d**) the microglial marker, ionized calcium binding adaptor molecule 1 (Iba1, green) in the dentate gyrus of (**a**, **b**) wildtype (WT) and (**c**, **d**) IGF1 overexpressing (IGFtg) mice at 6 weeks after injury. DAPI label is shown in blue. The scale bar represents 10 μm. GCL, granule cell layer; ML, molecular layer. (**e**) Density (cells/mm^3^) of BrdU+ cells within the ML was increased in both WT and IGFtg mice relative to their respective sham controls. Brain injury resulted in an increased density (cells/mm^3^) of (**f**) GFAP+BrdU+ cells and (**g**) Iba1 + BrdU+ cells within the ML relative to sham controls. IGF1 overexpression increased the relative area of (**h**) GFAP and (**i**) Iba1 immunostaining within the ML of injured mice compared to controls. Data presented as mean + SEM; *n* = 3 sham /genotype, *n* = 6–9 CCI /genotype. One-way ANOVA, followed by Bonferroni’s selected comparisons post-hoc t-tests: *p < 0.05 and **p < 0.01 compared to respective sham group

Experimental TBI is known to stimulate glial proliferation, producing long-lasting increases in numbers of astrocytes and microglia in the hippocampus. To examine whether IGF1 augmented trauma-induced gliosis, we quantified the numbers of cells generated within the first week after TBI within the ML of the DG, a region predominantly populated by glial cells, and identified glial phenotypes (astrocytes and microglia) of proliferated cells (Fig. 2a-d). As in the GCL, CCI brain injury was associated with a significant increase in the density of acutely proliferated (BrdU+) cells in the ML that persisted out to 6 weeks in both WT and IGFtg mice when compared to sham injury (Fig. 2e). Proliferation in the ML was not significantly altered by overexpression of IGF-1. Brain injury stimulated increases in both astrocyte (Fig. 2f) and microglia (Fig. 2g) proliferation, but the density of new glia surviving to 6 weeks post-injury was not significantly different in IGFtg mice as compared to WT mice. Injury-induced glial proliferation was also equivalent within the GCL (data not shown). While IGF1 overexpression did not increase numbers of new glia, the area of immunolabeling was significantly increased in the ML for both GFAP (Fig. 2h) and Iba1 (Fig. 2i) in IGFtg brain-injured mice when compared to sham controls. In sum, these data demonstrate that IGF1 strongly promotes the long-term survival of posttrauma-born hippocampal granule neurons, selectively increasing the density of new neurons without affecting the density of proliferated astrocytes or microglia in the DG after CCI brain injury.

### IGF1 enhances outward migration of posttrauma-born neurons in the GCL and attenuates ectopic localization of posttrauma-born neurons in the DG

**Fig. 3:**
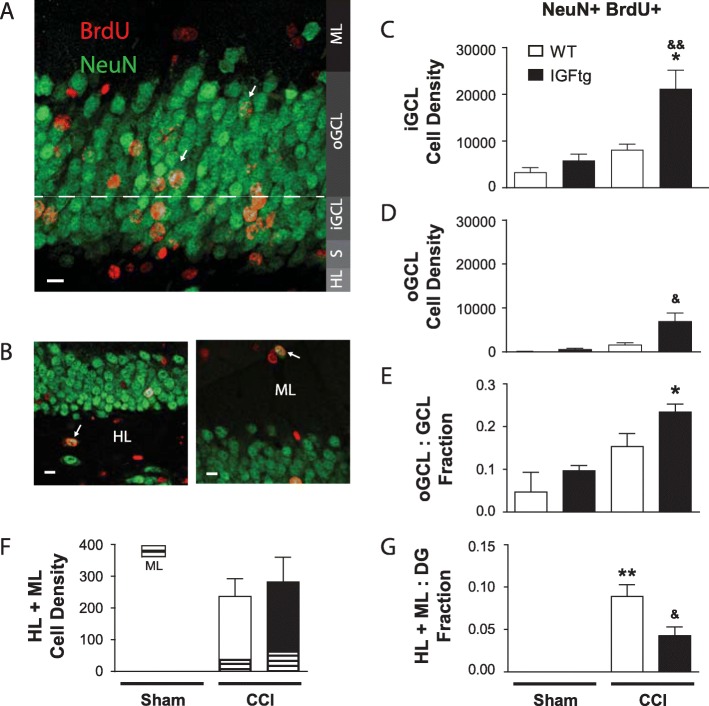
IGF1 decreases ectopic localization of new neurons within the dentate gyrus after injury. (**a**) Posttrauma-born mature neurons immunolabeled for Neuronal Nuclei (NeuN, green) and the proliferation reporter bromodeoxyuridine (BrdU, red) primarily localized to the inner granule cell layer (iGCL) and the outer 2/3rd region of the granule cell layer (oGCL, white arrows) after controlled cortical impact (CCI). S, subgranular zone. (**b**) Mature neurons (NeuN+BrdU+) were occasionally observed in the hilar layer (HL) and the molecular layer (ML) after CCI. White arrows highlight double-labelled cells; the scale bar represents 10 μm. IGF1 overexpression was associated with a significant increase in the density (cells/mm^3^) of matured post-trauma proliferated neurons in both the iGCL (**c**) and oGCL (**d**) at 6 weeks after brain injury. (**e**) The proportion of proliferated GCL neurons that localized to the oGCL was increased by IGF1 overexpression. (**f**) Brain injury stimulated the ectopic migration of a small subset of new neurons into the ML and HL in both injured WT and injured IGFtg mice. The ML and HL NeuN+BrdU+ densities (cells/mm^3^) are separated for illustrative purposes. (**g**) However, the proportion of acutely born neurons that matured and localized ectopically to the ML or HL was reduced by IGF1 overexpression. Data presented as mean + SEM; Sham n = 3/genotype, CCI *n* = 8–9/genotype; One-way ANOVA, followed by Bonferroni’s selected comparisons post-hoc t-tests. *p < 0.05 and **p < 0.01 compared to sham controls. &*p* < 0.05 and &&*p* < 0.01 compared to injured WT

Earlier studies that characterized the development and migration of newborn neurons within the GCL identified two different regions, the inner and outer zones [11, 61–64]. Adult-born neural progenitors proliferate in the SGZ and primarily localize to the inner 1/3 of the GCL (iGCL), while a few localize within the outer 2/3 of the GCL (oGCL) as they mature [11, 65]. The majority of neurons born the first week after surgery localized to the iGCL in all groups (Fig. 3a). Attendant with the 3-fold increase in new mature neurons in the GCL, IGF1 overexpression increased the density of mature posttrauma-born neurons localized to both the iGCL (Fig. 3c) and the oGCL (Fig. 3d) at 6 weeks postinjury when compared to WT mice. To determine if this increase in new neurons in the oGCL was merely reflective of the overall IGF-1-mediated increase in neurogenesis, we examined the relative proportions of posttrauma-born neurons localized to the oGCL. While brain injury did not increase the proportion of NeuN+BrdU+ neurons that localized to the oGCL in WT mice compared to WT sham controls, IGF1 overexpression modestly increased the proportion that localized to the oGCL (Fig. 3e).

We next sought to determine whether increased posttraumatic neurogenesis in IGF1 overexpressing mice was associated with heightened risk for ectopic localization of adult-born neurons. As illustrated in Fig. 3b, new neurons were occasionally observed within the HL and ML in brain-injured mice. While absent in sham-injured mice, ectopically localized new neurons (localized outside the GCL, i.e. ML + HL) were observed in both WT and IGFtg injured mice at a similar density (Fig. 3f; Student’s T-test, *p* = 0.636). Despite a 3-fold increase in the density of BrdU+ GCL neurons mediated by IGF1 (Fig. 1i), the proportion of posttrauma-born DG neurons that were ectopically localized outside the GCL following injury was significantly reduced by IGF1 overexpression (Fig. 3g).

### IGF1 improves reversal learning in RAWM 6 weeks after CCI

**Fig. 4:**
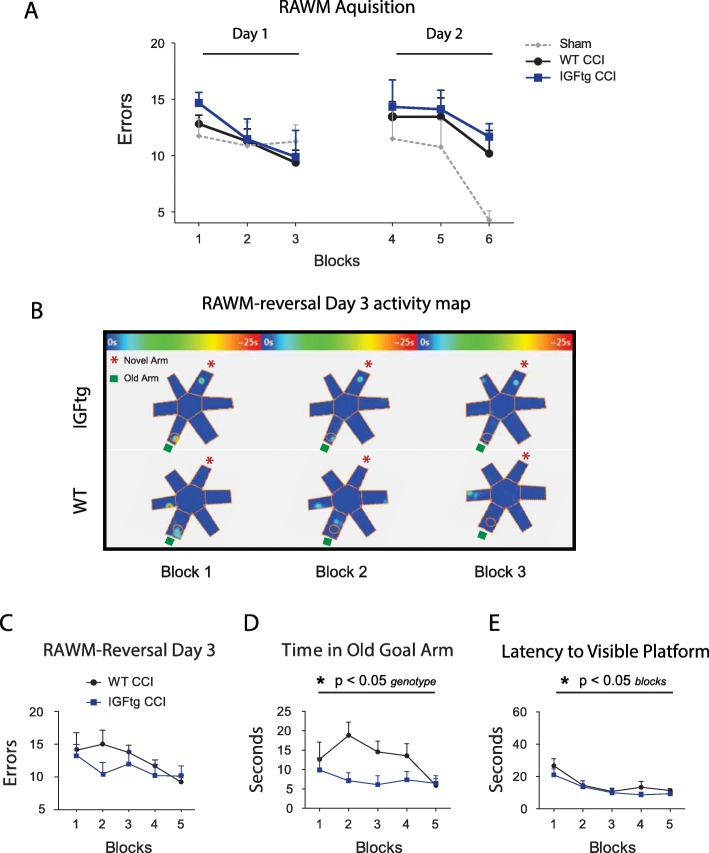
Brain-injured mice overexpressing IGF1 more rapidly learn to find a novel platform location. (**a**) Brain-injured wildtype (WT) and IGF1 transgenic (IGFtg) mice learned to find a hidden platform during radial arm water maze (RAWM) acquisition testing (Days 1–2). Both WT and IGFtg mice show a decrease in numbers of errors over training days. Sham control WT mice from a separate study are shown as a reference. (**b**) Heat maps illustrate activity patterns for representative IGFtg and WT mice during the first three blocks of reversal testing on Day 3 in the RAWM. IGFtg mice extinguished memory of the old goal arm sooner, after the platform was moved from the original training location (old arm; denoted by a green square) to a novel location (novel arm; denoted by red asterisk). Heatmap blue to red coloration indicates increasing amount of time. (**c**) Both WT and IGFtg mice show a decrease in numbers of errors during RAWM reversal testing. (**d**) WT mice consistently spent more time than the IGFtg mice in the previous platform location during RAWM reversal testing. (**e**) Injured WT and IGFtg mice have similar times to locate a visible platform at 6 weeks after CCI, confirming that differences between groups during the hidden platform trials were not due to sensory or motor dysfunction. CCI *n* = 9–11/genotype; Repeated measures ANOVA. *p < 0.05, main effect of genotype (in D) or block (in E)

To elucidate the effect of IGF1 overexpression on acquisition and reversal learning after injury, mice were evaluated in a 3d RAWM test. On acquisition days 1 and 2, the numbers of errors did not differ significantly between injured WT and IGFtg mice, indicating a similar ability to learn the initial platform location (Fig. 4a). During day 3 RAWM reversal testing, the platform was moved to a novel goal arm, requiring mice to change their strategy and identify the new platform location. While injured WT and IGFtg mice made similar numbers of errors initially on day 3 (block 1), injured IGFtg mice showed a rapid decrease in errors by block 2 while injured WT mice appeared to take several blocks of training before learning the new location (Fig. 4c). Activity maps from RAWM trials during the reversal learning phase illustrate that injured WT mice had more activity in the vicinity of the old goal arm in blocks 1, 2, and 3 than did injured IGFtg mice (Fig. 4b). A decrease in time spent in the old (original) location can indicate an animals’ ability to extinguish prior memory and learn a new strategy [20, 66]. Therefore, we quantified the time spent in the old goal arm during the reversal learning phase of RAWM testing. Injured IGFtg mice spent significantly less time in the old goal arm during day 3 of RAWM testing compared to injured WT mice (Fig. 4d). Both WT and IGFtg mice performed similarly on a visual platform test (Fig. 4e), indicating that differences in old arm exploration times are not due to motor or visual deficits [57, 67].

## Discussion

The fate, localization, and function of posttrauma-born neurons in the hippocampal DG 6 weeks following TBI were examined in IGF1 overexpressing and WT mice. Three key findings from the studies are presented. First, of the hippocampal cell types that proliferate after trauma, the population of posttrauma-born neurons that survive to maturity is selectively increased by elevated brain levels of IGF1. IGF1 overexpression significantly increased the number of mature posttrauma-born neurons but not the number of astrocytes or microglia that proliferated within the first week of injury. Second, we show for the first time that IGF1 regulates migration of adult-born neurons following trauma. Brain-injured IGF1 mice had significantly more newly born neurons localized deep within the GCL. Although IGF1 promoted outward migration within the GCL, it did not potentiate injury-induced ectopic granule cell localization to the HL and ML. Third, we present a novel effect of IGF1 on cognitive flexibility following trauma. At 6 weeks, injured IGFtg mice had improved performance on the day of RAWM reversal testing, when the hidden platform was moved to a novel location. This data compels further evaluation of IGF1 for its therapeutic potential to promote hippocampal neurogenesis and cognitive flexibility following trauma.

### Proliferation and survival

Brain injury drives extensive cell death of immature neurons in the GCL [22–24, 26]. Coincident with early neuron death, cellular proliferation increases markedly throughout the hippocampus, peaking around 3 days after TBI [27, 68, 69]. Proliferation in the ML and HL results predominantly in new glial cells, while the majority of proliferating cells within the GCL become neurons [23, 28, 32, 69]. Many groups have demonstrated, relative to sham controls, an increase in numbers of posttrauma-born GCL neurons which is sustained for up to 2 months after TBI, consistent with increased neurogenesis [23, 32, 34, 68]. However, it is unclear whether the milieu of the injured brain adversely affects the relative survival rate of newly born neurons. We show that, although TBI triggers an increase in cellular proliferation, the proportion of acutely proliferated cells that differentiate into neurons and survive to late-stage maturity is diminished by TBI in WT mice. The proportion of BrdU+ GCL cells that phenotype as mature neurons drops from approximately 60% in sham controls to less than 30% in WT mice 6 weeks after CCI, a decrease in close agreement with previous studies at 4–5 weeks after CCI [27] or lateral fluid percussion [23] TBI. Nonetheless, others have reported no change in the relative survival of posttrauma-born neurons in the GCL [28] or SGZ [70].

To examine the effect of IGF1 on proliferation and long-term survival of neurons born early after TBI, we utilized a transgenic mouse model in which IGF1 expression is driven through the GFAP promoter. We previously established that this construct leads to a significant elevation of IGF1 levels in the hippocampus as early as 1 day which increases further by 3 days after CCI brain injury, in parallel with robust hippocampal astrogliosis and upregulation of GFAP [48]. It is important to note that in sham controls, basal GFAP expression resulted in no detectable increase in human IGF1 levels. In the current study, no significant differences in proliferation or survival of posttrauma-born neurons or glia were found between WT and IGF1tg sham groups. Given the small number of mice available as sham controls, our current study was underpowered to detect small differences between sham groups; however, these findings are consistent with a larger study in which we also found no differences in early hippocampal proliferation or neurogenesis between IGFtg and WT sham controls (*n* = 8/genotype) [26].

By linking IGF1 expression to the GFAP promoter, IGF1 levels are amplified selectively in mice following CCI through posttraumatic astrogliosis [48]. Importantly, posttraumatic IGF1 overexpression stimulated a 3-fold increase in numbers of neurons that reached late-stage maturity 6 weeks after injury, restoring the proportion of proliferated cells that became mature neurons to a level equivalent to uninjured controls. IGF1 did not significantly enhance cellular proliferation, but may promote increased generation of mature neurons by promoting neuronal differentiation and attenuating apoptosis.

Our previous work demonstrated that IGF1 overexpression resulted in an increase in neuronal differentiation of SGZ neural stem cells and greater immature neuron dendritic complexity within 10 days of CCI brain injury [26]. We and others have demonstrated that posttrauma-born GCL neurons develop with abnormal, stunted dendritic arborization [26, 33, 71]. Improved dendritic complexity may protect against apoptosis during synapse-dependent competitive survival of adult-born neurons [8–11]. In vitro studies and developmental studies suggest that IGF1 supports the survival of newborn neurons to end-stage maturity through cooperative inhibition of programmed cell death and enhanced immature neuron dendritic arborization [45, 48, 72–76]. It is not yet known whether IGF1 overexpression decreases the rate of apoptosis of newly proliferated neurons in the GCL. However, IGF1 overexpression has been shown to protect against hilar neuron and CA3 pyramidal neuron loss after TBI [48].

IGF1 overexpression facilitated the long-term survival of posttrauma-born neurons without increasing numbers of new astrocytes or microglia, suggesting a selective effect on neuronal survival. Our data corroborates previous studies describing acute proliferation and long-term survival of glial cells in the ML after injury [32]. It is possible, then, that the selective effect of IGF1 on newborn neuronal survival may be due in part to inhibition of apoptosis pathways that are activated after TBI to a greater extent in neurons as compared to glia [77]. Because the IGF1 receptor is ubiquitously expressed on all neural cell types [41, 78], it would be expected that astrocyte-derived IGF1 overexpression affects multiple cell types. IGF1 appeared to act on both astrocytes and microglia, potentiating injury-induced hypertrophy, as suggested by increases in the area of labelled glia. While we previously showed acutely elevated hippocampal IGF1 levels and an IGF1-mediated increase in astrocyte hypertrophy in IGFtg mice after CCI brain injury [48], it is not yet known whether the chronic astrocyte hypertrophy noted in the current study supports increased in brain IGF1 levels driven through the GFAP promoter as long as 6 weeks post-injury.

### Migration

In naïve animals, the majority of adult-born neurons migrate from the neurogenic niche of the SGZ within the first 4 weeks of their birth and remain within the iGCL [11, 79, 80]. Consistent with previous reports [33, 34, 38], we demonstrate that TBI promotes a modest increase in the number of posttrauma-born neurons that migrate further outward to the oGCL. The functional implications of this enhanced migration are not yet clear. Posttrauma-born GCL neurons appear to synaptically integrate within the DG [34] and connect to appropriate CA3 targets [69, 81]. However, newly matured neurons located in the oGCL exhibit more pronounced deficits in dendritic arbor development even 5 weeks post-trauma [33]. Here we show that, in addition to improving dendritic arborization of immature neurons following injury [26], IGF1 regulates outward migration of posttrauma-born neurons, consistent with a developmentally born phenotype. IGF1 regulates known factors of neuronal migration including GSK3B, PSA-NCAM, Cdk5 and reelin [12, 44, 82]. Signalling studies have revealed that inhibiting the activity of GSK3B, a protein inactivated downstream of IGF1 signalling, is sufficient to increase the number of adult-born neurons localized to the oGCL [83]. This may be an indication that IGF1 can accelerate development of newborn neurons following trauma.

As immature neurons develop in the postnatal DG, they are rarely found localized to regions outside the GCL. In the uninjured brain, during the process of adult neurogenesis, immature DG granule neurons that ectopically localize within the DG undergo cell-mediated apoptosis before they reach maturity [84, 85]. Although quantification of newborn neurons is often restricted to the SGZ and GCL [23, 86], ectopic migration to either the ML [33, 34, 39] or the HL [33, 40] has been reported after experimental TBI. Although a relatively small number of posttrauma proliferated neurons localize ectopically to the HL and ML at 6 weeks post-CCI in WT and IGFtg mice, their physiological relevance remains unclear. Ectopic hilar neurons are thought to contribute to hippocampal hyperexcitability [87, 88] and seizure development [89, 90]. Furthermore, the ectopic localization of immature adult-born granule neurons associated with the neuropathology induced by epilepsy suggests a contribution to maladaptive plasticity [89, 91]. The CCI model of brain trauma does produce delayed mossy fiber sprouting and seizure activity, particularly with more severe injuries [92, 93]. While we did not observe seizure behaviour in injured mice, our study was designed to examine posttraumatic hippocampal neuroplasticity that develops prior to the window of seizure onset, which is estimated to be as late as 8 weeks after CCI [92].

While key factors regulating posttraumatic newborn neuron migration are not fully known, IGF1 may play a role in inhibiting ectopic hilar migration. We found that IGF1 overexpression inhibits the localization of mature posttrauma-born granule neurons to regions outside the DG GCL. IGF1 may regulate immature neuron migration indirectly through an effect on reelin, a migratory stop signal expressed by hilar basket cells [85] which prevents granule cells from migrating ectopically into the hilus [94]. Increasing neurogenesis after TBI has been suggested to enhance the likelihood of seizure activity [86]; however, IGF1 treatment has been shown to reduce seizure behavior in a rodent model of temporal lobe epilepsy [75].

### Reversal learning

Systemic IGF-1 administration and astrocyte-specific IGF1 overexpression have both been shown to improve cognitive function within the first 2 weeks after TBI [47–49]. Our study provides the first evidence that increasing brain levels of IGF1 following injury can influence cognition in the chronic phase after TBI, using a reversal water maze task shown to be sensitive to alterations in hippocampal neurogenesis. At 6 weeks after TBI, during the reversal phase of a RAWM test when the hidden platform was moved to a new location, injured IGFtg mice spent less time in the original goal arm than did injured WT mice. Inhibition of adult neurogenesis using the anti-proliferation drug temozolomide caused a strong and lasting preference for the old goal arm during Morris water maze testing when the goal arm was moved to a novel location [20]. Thus, it is possible that injured WT mice had an increased preference for the original goal location due to impaired neurogenesis. Conversely, injured IGFtg mice exhibited significant enhancement of posttraumatic hippocampal neurogenesis with concomitant improvement in RAWM reversal learning. Increasing the number of mature adult-born neurons is sufficient to improve the integration of novel information upon introduction of a novel platform location during water maze assessment in mice, demonstrating cognitive flexibility and memory extinction [14, 15, 18, 95].

Acquisition learning may be less sensitive than reversal learning to modulation of hippocampal neurogenesis, as suggested by previous studies demonstrating that an attenuation of adult neurogenesis does not prevent acquisition of the escape platform location during water maze training [20, 96]. Brain-injured WT and brain-injured IGF overexpressing mice showed equivalent abilities to learn the location of a hidden platform during the acquisition phase (days 1–2) of RAWM testing. Although we and others have demonstrated that moderate or severe CCI results in persistent learning and memory dysfunction in mice [97–102], it is possible that the lack of effect of IGF-1 during acquisition may be related to an inadequate behavioral dysfunction at 6 weeks postinjury.

Our data show that increasing brain levels of IGF1 enhances hippocampal plasticity by promoting maturation and long-term survival of neurons born after TBI and improving cognitive flexibility. These findings have important implications for treatment of TBI-related cognitive dysfunction. Over 65% of patients with moderate to severe TBI report long-term cognitive deficits [103]. Modulation of adult hippocampal neurogenesis is a potential therapeutic target for TBI recovery. Collectively our findings support IGF1 supplementation following brain injury as a potential means to enhance appropriate and persistent posttraumatic hippocampal neurogenesis.

## Conclusions

This work is critical to our understanding of adult-born neuron development in the context of brain injury. Stimulation of neural stem cell proliferation by TBI contributes to recovery of the vulnerable immature hippocampal neuron population, but the extent of long-term survival and functional integration of neurons born in the aftermath of trauma is debated. Further, TBI alters the morphological development and localization of posttrauma-born neurons, raising concerns about detrimental consequences of implementing therapies that augment posttraumatic neurogenesis. We show that IGF1 (a) promotes acute neurogenesis without increasing gliogenesis, (b) enhances migration within the GCL but decreases aberrant migration outside the GCL, (c) supports end-stage maturity of newborn neurons, and (d) promotes hippocampus-dependent cognitive flexibility. These data support the continued evaluation of IGF1 as a potential therapeutic for neurological disorders and disease.

## Data Availability

The datasets used and/or analysed during the current study available from the corresponding author upon reasonable request.

## Abbreviations

ANOVA: Analysis of variance
BrdU: Bromodeoxyuridine
CA3: Cornu ammonis field-3 of the hippocampus
CCI: Controlled cortical impact
Cdk5: Cell division protein kinase 5
CNS: Central nervous system
DAPI: 4′,6-diamidino-2-phenylindole
DG: Dentate gyrus
dpi: Days post-injury
GCL: Granule cell layer
GFAP: Glial fibrillary acidic protein
GSK3B: Glycogen synthase kinase 3 beta
HL: Hilar layer
Iba1: Ionized calcium-binding adapter molecule 1
iGCL: Inner 1/3 of the GCL
IGF1: Insulin-like growth factor-1
IGFtg: IGF1 double transgenic mice
ML: Molecular layer
NeuN: Neuronal nuclei
NHS: Normal horse serum
oGCL: Outer 2/3 of the GCL
PSA-NCAM: Polysialylated neuronal cell adhesion molecule
RAWM: Radial arm water maze
ROI: Region of interest
SEM: Standard error of mean
SGZ: Subgranular zone
TBI: Traumatic brain injury
TBS: Tris-buffered saline
WT: Wildtype
